# Dissecting the Circle of Willis-Migraine connection: A review

**DOI:** 10.3934/Neuroscience.2025001

**Published:** 2025-02-11

**Authors:** Jad El Choueiri, Leonardo Di Cosmo, Francesca Pellicanò, Francesca Romana Centini

**Affiliations:** Humanitas University, Department of Biomedical Sciences Via Rita Levi Montalcini, 4, 20072 Pieve Emanuele Milano, Italy

**Keywords:** Circle of Willis, migraine with aura, migraine without aura, cerebral vascularization

## Abstract

**Objective:**

Anatomical variations in the Circle of Willis (CoW) may mediate the prevalence of migraines with aura (MWA) and without aura (MWoA) in patients. The aim of this review is to describe and evaluate contrasting studies to clarify the current understanding of this association within the literature.

**Methods:**

A comprehensive search across PubMed, Google Scholar, and the Cochrane Library resulted in 10 relevant studies that met our selection criteria and examined the association between the CoW and migraine prevalence.

**Results:**

Conflicting results were reported across the prospective and retrospective studies, which varied among different populations and the inclusion classification of CoW variants. Studies that evaluated posterior CoW variations repeatedly reported differential associations between migraines with aura (MWA) and without aura (MWoA), thus revealing a significant association only with the former. Two mechanisms of actions were hypothesized to be attributed to such associations; one hypothesized a resultant cerebral hypovascularization, whilst the other emphasized the role of shear stress in associated small arteries.

**Discussion:**

While some studies reported significant associations between specific CoW variations and migraines, particularly with the posterior CoW variations and MWA, conflicting evidence emphasizes the necessity for further investigations to provide a greater understanding between CoW variations and different migraine subtypes. A consensus calls for future studies to include larger samples over various ethnic populations to overcome the biases encountered within the current field of literature.

## 1. Introduction

A migraine, a complex neurological disorder, is characterized by recurrent attacks of moderate to throbbing headaches, nausea, and photosensitivity, that may or may not be accompanied by other neurological symptoms [1]. The two main types that have been explored are migraines with aura (MWA) and migraines without aura (MWoA); the former presents neurological symptoms prior to onset, whilst the latter and more common form occurs without warning. The complex pathomechanism and contributing factors of migraines remain incompletely understood, with recent studies underscoring the potential link between cerebrovascular alterations through specific Circle of Willis (CoW) variations and migraine development [2]–[4].

The CoW is a network of arteries at the base of the brain responsible for supplying the brain with arterial blood and for collateral blood flow to cerebral tissue, and it is highly variable by nature [5]. These variations have been associated with alterations in cerebral blood flow (CBF) [6]. Such hemodynamic changes have been attributed to hemorrhagic strokes [7] and ischemic strokes [8], though their association with the pathogenesis of migraines remains unclear.

A review of the literature shows that a consensus on the nature of the link between CoW variations and migraines has not been reached yet; some studies have found significant associations, whilst some others have found none, though there appears to be a differential association with MwA compared to MwoA. In an attempt to clarify the state of the art, we analyzed the current literature and summarized the findings.

## 2. Materials and methods

To explore the relationship between CoW variations and migraines, an extensive search of the available literature was conducted through databases including PubMed and Cochrane. The search focused on studies published in English, and applied a range of synonyms surrounding the topics of migraines and the CoW including but not limited to the following: “Circle of Willis”, “Circle of Willis variations”, “Migraine with aura”, “Migraine without aura”, “Cerebral blood flow”.

The articles were selected based on their relevance to the topic, with studies not directly addressing this association being excluded. A systematic approach was used to organize and extract data, with findings grouped thematically to reflect their contributions to specific aspects of the CoW-migraine association.

## 3. Results

The potential link between CoW variations and the development of migraines is a topic of ongoing debate. While some studies support the association between the two, others claim to have found no association.

### 3.1. Associating anatomical definition and migraine predisposition

Several studies have investigated this potential link. For instance, Cavestro et al. [9] categorized anomalies as any variation discrepant to a complete circle (stenosis, hypoplasia, aplasia) and reported increased anatomical variations within the CoW in migraineurs against the control group. Specifically, they conducted a cohort case-control study (n = 429 participants; of which 270 migraineurs and 159 controls), and found that migraineurs presented an anatomical variant in 108 (40%) cases, whilst 34 participants of the control group (21.4%) presented a variant. A significant association was found between MWoA and variants [Odds Ratio (OR) = 2.4, 95% CI (1.5 to 3.9)] and between MWA and variants [OR = 3.2, 95% CI (1.6 to 4.1)]. They further reported a significant association between the prevalence of unilateral posterior variants in basilar hypoplasia with MWA as compared to the controls [OR = 9.2, 95% CI (2.3 to 37.2)].

To add a further layer of distinction, many studies investigated more specific variations and their association with migraines. For instance, Bugnicourt et al. [10] classified variations in their study as anatomical deviations within the posterior CoW system, whether it be stenosis, hypoplasia, or aplasia of the posterior communicating arteries (PCoA) or posterior cerebral arteries (PCA). The study evaluated the prevalence of MWA and MWoA in 124 patients with either the presence or absence of posterior CoW variations and found a significant relation (p < 0.001) for an incomplete posterior CoW in migraineurs. No significant difference was found between the migraineur groups with and without auras.

In a similar manner, Ibrahim et al. [11] also found a significant association between the presence of migraines and an incomplete posterior circle (p = 0.002) in a cohort of 47 patients in Sudan; additionally, they suggested bilateral hypoplasia of the PCoA as the most prevalent variation associated with high-frequency migraine attacks.

Alternatively, some studies proposed an association between migraines and anterior variations of the CoW. This alternative categorization of CoW variations places emphasis on the stenosis, hypoplasia, and aplasia of the anterior communicating artery (ACoA). In this framework, a cross-sectional study by Dixit et al. [12] evaluated 132 patients and described the frequency of the absence of AcoA being greater in migraine patients than non-migraine patients (p value = 0.04329)* (*No p-value was provided by the authors of the paper, therefore a Chi-squared test was conducted with the available data provided).

### 3.2. Migraine with and without aura: a differential association?

Many studies explored the differential association between CoW variations and migraine presentation, thereby highlighting the differential association between specific variants and the prevalence of MWA and MWoA. Such was exemplified by Cavestro et al. [9], who found that posterior anomalies were more frequent in patients who suffered from migraines with aura, citing evidence of hereditary migraines associated with inherited CoW variants [13] and highlighting the disease's likely multifactorial etiology.

Cucchiara et al. [3] also observed a significant association between the presence of incomplete CoW in patients with MWA vs. the control (p = 0.02) in a cohort of 170 patients, with no significant difference in patients with MWoA compared to the control (p = 0.08). Additionally, the study observed the changes in hemispheric blood flow and its association with variations of the CoW structure. Notably, they found that posterior cerebral artery variants were significantly associated with the greatest asymmetry of blood flow (p = 0.02), thus suggesting that such a factor could contribute to migraine pathogenesis.

Henry et al. [14] validated these findings in a meta-analysis, thus suggesting a stronger association between migraines and an incomplete posterior circle (OR = 2.60, 95% CI 1.79–3.76, p < 0.00001) compared to an incomplete anterior circle (OR = 2.01, 95% CI 1.15–3.53, p = 0.01). Additionally, they found a higher prevalence of migraines in patients with variants compared to those with complete circles (OR = 2.27, 95% CI 1.53–3.38, p < 0.0001), though their criteria that defined normalcy was not clearly specified. Notably, their analysis emphasized the significant association between migraines with aura and an incomplete posterior circle, thus corroborating hypotheses proposed in earlier studies (OR = 2.10, 95% CI 1.39–3.17, p = 0.0004).

### 3.3. Studies reporting no link: A call for further investigation

Not all studies support this association. For instance, in a retrospective study that included 85 female patients, Ezzatian-Ahar et al. [15] found no significant difference between the prevalence of incomplete CoW in migraineurs vs the control (p = 0.252). Similarly, in a population of acute stroke patients, Hamming et al. [16] observed no difference in the completeness of the CoW versus the controls.

### 3.4. Proposed mechanisms of action

Among the studies that found a significant association, many tried to hypothesize the mechanisms that relate the CoW variations to migraine pathogenesis. Borgdorff et al. [4] hypothesized a mechanism attributing the shear stress within the cerebral vasculature to the pathogenesis of migraines. This proposition suggests that CoW variations mediate the hemodynamic flow of blood through the vessels themselves, thereby acting as a possible inducer of cerebrovascular damage through increased wall shear-stress, especially in small-diameter anastomotic vessels. Such was proposed to lead to endothelial dysfunction, platelet aggregation, and potential aneurysm formation, which may therefore predispose patients to a migraine onset. Recent studies that observed the hemodynamics of CoW through transcranial doppler imaging found significant associations between migraines with aura, vessel pulsatility, and blood flow in several arteries of the CoW [17], though a further investigation remains necessary.

In contrast, Cavestro et al. [9] argues that CoW variations may contribute to changes in cortical vasculature. As hypothesized in the study, the decline of cortical vascularisation, specifically as regards posterior CoW malformations, may stimulate neuro-vascular cortical spreading depression, which, in turn, may contribute to the pathophysiology of migraines with aura [3].

## 4. Discussion

The current body of research investigating the potential link between CoW variations and migraines presents a complex picture. Studies that explored CoW variations in MWA patients provided compelling evidence for potential associations, while the association between CoW variants and MWoA is comparatively weaker, even among studies that suggested their positive associations. Notably, only two studies reported absolutely no association between CoW variations and migraine development [15],[16].

Generally, the literature surrounding CoW variations and migraine development demonstrates a significant heterogeneity. The categorization of the physiologically normal CoW morphology varied significantly between studies. This could contribute to explaining the discrepancy between the statistical frequencies of incomplete CoWs between the control groups studied and other statistical reports using larger samples of healthy subjects [18]. For example, Cavestro et al. [9] employed a more holistic variant classification, thereby evaluating features from vessel diameter to displacement, potentially producing an overestimation within the variant group. Contrastingly, Bugnicourt et al. [10] neglected measuring the vessel diameter and position, but rather evaluated their presence or absence, thereby only taking record of incomplete CoW formations.

Furthermore, a lack of standardized reference measurements of normal vessel diameters complicates comparisons between studies, potentially accounting for differential reports of stenotic and hypoplastic malformations. For example, Cucchiara et al. [3] and Ezzatian-Ahar et al. [15] defined hypoplastic arteries in the CoW as having a diameter less than 0.8 mm in accordance with the scheme published by Krabbe-Hartkamp et al. [18], whilst Hamming et al. [16] defined abnormalities as vessels with a diameter of less than 1.0 mm. These discrepancies within definitions may account for conflicting results.

Such discrepancies between populations were also exemplified in the study conducted by Cavestro et al. [9], where they measured the basilar artery diameter to potentially account for a sub-group of posterior CoW malformations. Upon the result analysis, they reported the mean diameter as 3.5 mm, which deviated from a reference study conducted on a larger cohort, measuring an average of 3.0 mm [18]. This may possibly be accounted for by the genetic component of CoW formations in different ethnic populations alongside the presence of a small sample size.

Additionally, the generalizability of findings in CoW variation research is often constrained by limitations in the sample size. For instance, Cucchiara et al. [3] reported no significant association between MWoA and posterior CoW variations, though the relatively small sample size may affect the study's statistical power and applicability. This was further emphasized by the fact they reported a decreased prevalence of variations in females in both the migraineurs and the control groups, while Klimek-Piotrowska et al. [19] reported a contrasting increased prevalence of variations in both female groups. These conflicting results emphasize the necessity for larger studies to achieve more definitive and generalizable conclusions.

This heterogeneity among the included studies, as discussed above, is synthesized in Table 1, which provides a descriptive summary of the study designs, sample sizes, reporting parameters, and relevant findings.

**Table 1.** Summary of the included studies.

**Title**	**First Author**	**Year of publication**	**Study design**	**Sample size**	**Findings (%)**
Incomplete posterior circle of willis: A risk factor for migraine?: Research submission	Bugnicourt J	2009	Retrospective study	Patients = 124 MWA = 24 MWoA = 23 Controls = 77	***MwA:*** Anterior incomplete = 8.7 Posterior incomplete = 60.9 COW incomplete = 65.2 ***MwoA:*** Anterior incomplete = 4.2 Posterior incomplete = 37.5 COW incomplete = 37.5 ***Controls:*** Anterior incomplete = 5.2 Posterior incomplete = 18.2 COW incomplete = 22.1 Fetal configuration = 14.3
Anatomical variants of the circle of willis and brain lesions in migraineurs	Cavestro C	2011	Retrospective study	Patients = 429 MWA = 66 MWoA = 204 Controls = 159	***MwA***: Normal COW = 54.6 Anterior variant = 9.1 Posterior variant = 34.8 Both = 1.5 Variant COW = 45.5 ***MwoA:*** Normal CoW = 61.8 Anterior variant = 9.8 Posterior variant = 23.5 Both = 4.9 Variant CoW = 38.2 ***Controls:*** Normal CoW = 78.6 Anterior variant = 5.0 Posterior variant = 13.2 Both = 3.1 Variant COW = 21.4
Migraine with Aura Is Associated with an Incomplete Circle of Willis: Results of a Prospective Observational Study	Cucchiara B	2013	Retrospective study	Patients = 170 MWA = 56 MWoA = 61 Controls = 53	***MwA:*** Anterior incomplete = 32.0 Posterior incomplete = 64.0 CoW incomplete = 74.0 Fetal PCA = 30.0 ***MwoA:*** Anterior incomplete = 21.0 Posterior incomplete = 57.0 CoW incomplete = 67.0 Fetal PCA = 26.0 ***Controls:*** Anterior incomplete = 13.0 Posterior incomplete = 41.0 CoW incomplete = 51.0 Fetal PCA = 34.0
Migraine without aura is not associated with incomplete circle of Willis: a case–control study using high-resolution magnetic resonance angiography	Ezzatian-Ahar S	2014	Retrospective study	Patients = 84 MWoA = 48 Controls = 37	***MwoA:*** CoW incomplete = 43.0 ***Controls:*** CoW incomplete = 41.0
Association of migraine headaches with anatomical variations of the circle of willis: Evidence from a meta-analysis	Henry B	2015	Meta-analysis	***Across four studies*** Patients = 807 MWA = 435 MWoA = 661 Controls = *not specified for each study*	***MwA:*** Anterior incomplete = 32.0 Posterior incomplete = 64.0 CoW incomplete = 73.0 ***MwoA:*** Anterior incomplete = 21.0 Posterior incomplete = 57.0 CoW incomplete = 67.0 ***Controls:*** Anterior incomplete = 13.0 Posterior incomplete = 41.0 CoW incomplete = 51.0
Abnormal Circle of Willis among Migraineurs in Sudan	Ibrahim S	2017	Retrospective study	Patients = 147 PWM (*MwA and MWoA not specified*) = 47 Controls = 100	***Migraineurs***: Normal configuration = 15.0 Incomplete configuration = 59.5 Hypoplastic configuration = 44.6 Fetal type = 6.4 Defective CoW = 85.0 ***Controls***: Normal configuration = 32.0 Incomplete configuration = 53.0 Hypoplastic configuration = 9.0 Fetal type = 6.0 Defective CoW = 68.0
Circle of Willis variations in migraine patients with ischemic stroke	Hamming A	2019	Prospective study	Patients = 646 (***stroke patients***) PWM = 52 MWA = 29 MWoA = 23 Controls = 594	***MwA:*** Anterior incomplete = 21.0 Posterior incomplete (unilateral = 24.0) Posterior incomplete (bilateral = 52.0) Overall CoW incomplete = 79.0 ***MwoA:*** Anterior incomplete = 9.0 Posterior incomplete (unilateral = 39.0) Posterior incomplete (bilateral = 57.0) Overall CoW incomplete = 96.0 ***Controls:*** Anterior incomplete = 10.0 Posterior incomplete (unilateral = 31.0) Posterior incomplete (bilateral = 54.0) Overall CoW incomplete = 85.0
Incidence of hypoplastic posterior communicating artery and fetal posterior cerebral artery in Andhra population of India: a retrospective 3-Tesla magnetic resonance angiographic study	Bhanu SP	2020	Retrospective study	Patients = 231 *(**all migraineurs**)*	***Hypoplastic PcoA:*** Overall incidence = 27.3 Right-sided hypoplasia = 6.9 Left-sided hypoplasia = 4.3 Bilateral hypoplasia = 16.0 ***Fetal PCA:*** Overall incidence = 5.6 Right-sided = 3.0 Left-sided = 2.6
Transcranial doppler evaluation of the cerebral vasculature in women patients who have migraine with Aura	Petrušić I	2020	Cross-sectional study	Patients = 103 MWA = 54 Controls = 49	***MwA:*** Increased PI in all segments of left and right MCA and ACA = ***significant*** BHI on both sides = ***significant*** Lower MV in the right vertebral artery and first segment of the basilar artery = ***significant Controls:*** Baseline PIs, BHIs and MVs = ***normal for comparison***
Detailed study of arterial variations in circle of Willis among migraine and non migrainous patients in Western Uttar Pradesh	Dixit V	2022	Cross-sectional study	Patients = 132 PWM = 58 Controls = 74	***PwM:*** Normal CoW = 67.2 Anterior incomplete = 26.7 Right posterior incomplete = 1.3 Left posterior incomplete = 1.35 COW incomplete = 32.8 ***Controls:*** Normal CoW = 75.7 Anterior incomplete = 12.2 CoW incomplete = 24.3

Note: PI: Pulsatility Index; MCA: middle cerebral artery; ACA: anterior cerebral artery; BHI: breath-holding index; MV: mean velocity; MWA: migraine with aura; MWoA: migraine without aura; PWM: patient with migraine.

Furthermore, the prevalence of CoW variations among different ethnic populations highlights the importance of the genetic component in cerebrovascular anatomy. Studies have revealed the significant changes in the distributions of CoW variations between various ethnic groups [20]. This further emphasizes the necessity for multicultural studies to generate sets of standardized values to define CoW variants and abnormalities.

The presence of CoW variants is not the sole determinant of changes in cerebral blood flow. The cerebrovascular compensatory capacity of different individuals varies significantly with different CoW presentations, and the intracranial collateral circulation differs among individuals [21]. Though many studies we evaluated inferred that the cerebrovascular changes were induced by variations in the CoW, such collateral intracranial compensation was not directly measured.

Additionally, age is an important consideration in cerebrovascular analyses, as younger patients were reportedly more likely to have a complete CoW [15],[18], the incidence of a complete circle decreases with age for both genders, and the presence of hypoplastic vessels significantly increases [22]. Sex-linked differences are also evident; recent meta-analyses have shown that females exhibit a higher prevalence of complete CoW than males [23], thus corroborating the previously established literature [3],[18]. However, a separate large cohort study of 1864 participants not included within this review reported no significant sex differences in the prevalence of the complete CoW variant [5]. This discrepancy was attributed to variations in CoW classification systems, thus emphasizing the need for a metric for standardization [23]. This disparity has been a topic for controversy, as the increased prevalence of complete circles conflicts with the higher proportion of migraines. Recent literature has drawn a parallel between the association between certain intracranial aneurysms in women, which may relate to CoW variations and their influence on hemodynamic stress [23]. For instance, hypoplastic or absent anterior cerebral arteries in men have been linked to an increased contralateral blood flow and the formation of ACoA aneurysms [24]–[26], while fetal-type posterior CoW variants in women are associated with internal carotid artery (ICA) aneurysms [27]. Whether these sex-specific hemodynamic mechanisms also predispose individuals to migraines remains unclear and warrants further investigations in parallel to other theories regarding hormonal influences and other physiological factors unique to each sex [23].

Aside from sex, age, and race variations, the classification of subjects as “headache-free” presents a possible limit, though they may develop migraine later in life, as many time-dependent factors might influence the CoW-migraine link. From there, future research should look beyond a static picture and capture the evolving interplay between migraine development and CoW variations.

Studies employed specific methods for participant acquisition that may introduce selection bias. Many studies recruited patients from neurological clinics, which may produce an unrepresentative control group. Thus, clinic centered populations may overrepresent individuals with severe symptomatic CoW variations whilst underrepresenting milder migraine presentations. The considered patients would be likely to suffer from a higher frequency, intensity, or duration of migraine attacks, not accounting for other pathologies they might suffer from. For example, Ibrahim et al. [11] recruited a group of participants composed of medical staff and volunteers that underwent a non-rigorous screening process. This approach introduces concerns about potential selection bias, due to their differential access to healthcare and health awareness in respect to the general population, therefore being a source of potential overestimation of the association in question. This highlights the need to consider population variability in CoW variations and its potential impact on the prevalence of migraine presentations.

Moreover, the contrasting results may be a product of specific methodological limitations within each study.

For instance, in a retrospective study, Ezzetian-Ahar et al. [15] found no significant association between CoW variations and MWoA. The cohort consisted solely of female patients from a single neurological clinic, thus producing a potentially unrepresentative sample, and the grouping of CoW variations as generalized deviations from a complete structure may have masked potential subgroups within the sample.

This limitation alongside the retrospective design used in this and other studies may introduce bias in patient selection and affect the identification of potential prevalences between CoW variations in migraineurs.

Similarly, Hamming et al. [16] looked at CoW variations in migraine patients with ischemic strokes utilizing a self-reported questionnaire to assess the subjects' past experiences, thus inducing potential recall bias. Given the specific vascular nature of the pathology, the inclusion of patients who previously suffered a stroke in the sample may potentially introduce confounding variables.

Bugnicourt et al. [10] may have conducted an insufficient participant exclusion in the control group, including patients with other neurological deficits, alongside hosting a predominant female patient population.

Furthermore, Cavestro et al. [9] had a non-selected control group, thereby selecting patients undergoing an MRI with an absence of migraines, and failed to account for extraneous variables introduced with comorbidities.

We conducted a further analysis to assess whether the statistical approaches used were appropriate. The goal was to determine if the chosen methods could have introduced bias into the results.

In terms of the data comparison, the Student's T-Test is markedly used among the reviewed papers [3],[11],[16],[28], along with the ANOVA test [9],[11],[28] and the Wilcoxon ranked-sum test/Mann-Whitney U test [3],[16].

To examine the association between categorical items and other data, the Chi-Square is the most commonly used approach [3],[9],[12],[16],[28]. Ezzatian-Ahar et al. [15] utilized a Multiple Logistic Regression Analysis in the presence of a categorical dependent variable and one or more explanatory variables. Along with univariate analysis, Bugnicourt et al. [10] applied a multivariate stepwise regression analysis to identify the most significant variables associated with a specific outcome.

Overall, the proposed statistical approaches appear to be appropriate and well-suited to their specific purposes. Therefore, we suggest that any discrepancy in conclusion across the studies is not related to the chosen statistical methods, but rather derived from the other factors previously discussed. Thus our review remains restricted by the limitations of the studies discussed.

The complexity of this potential link may be attributed to a multitude of factors; the heterogeneity of both CoW variations and the migraine phenotypes must be discussed. The greatly varying CoW presentation and severities, coupled with diverse presentation of migraine subtypes, make it challenging to quantify consistent associations. Alongside this, extraneous environmental factors mediate this association such as stress, sleep disturbances, hormonal fluctuations, and dietary triggers [29], all of which should be taken into account and potentially explored.

The establishment of such an association could potentially serve as a marker for the identification of individual predispositions for migraines with or migraines without aura, potentially guiding personalized treatment approaches and stimulating the development of novel therapeutic [3]. The study by Cucchiara et al. [3] underscores how the identification of structural alterations in the cerebral vasculature of migraine patients would provide a developmental mechanism for migraine susceptibility, giving insights into the genetic predisposition for migraines. They went further to mention the importance of understanding the potential mechanism underlying migraine aura and linked it to cerebral infarctions. Understanding these associations may even help to identify patients at risk of progressive cerebral ischemia and migraine pathogenesis. The researchers used the analogy to the current paradigm in ischemic strokes, in which the determining stroke mechanism is critical to therapeutic decision-making.

Despite certain advancements, significant gaps in knowledge remain, calling prospective studies that incorporate larger and more diverse populations to action, going deeper into the interplay between CoW variations, genetics and environment. Additionally, understanding the plasticity of the CoW, as observed in cases of flow diversion, could also inform future research on its role in the compensatory mechanisms underlying migraines [30]. Such a multifaceted approach would clarify the picture of the CoW-migraine link.

## 5. Conclusions

In conclusion, the association between CoW variations and migraines, particularly MWA, has been a topic of interest. While some studies have reported significant associations between specific CoW variations and migraines, particularly with the posterior CoW variations and MWA, conflicting evidence emphasizes the necessity for further investigations to provide a greater understanding between CoW variations and different migraine subtypes.

## Use of AI tools declaration

The authors declare they have not used Artificial Intelligence (AI) tools in the creation of this article.
